# Coordinate-Free and Low-Order Scaling Machine Learning Model for Atomic Partial Charge Prediction for Any Size of Molecules

**DOI:** 10.1021/acs.jcim.4c00376

**Published:** 2024-05-17

**Authors:** Qin Xie, Andrew P. Horsfield

**Affiliations:** Department of Materials, Imperial College London, SW7 2AZ London, U.K.

## Abstract

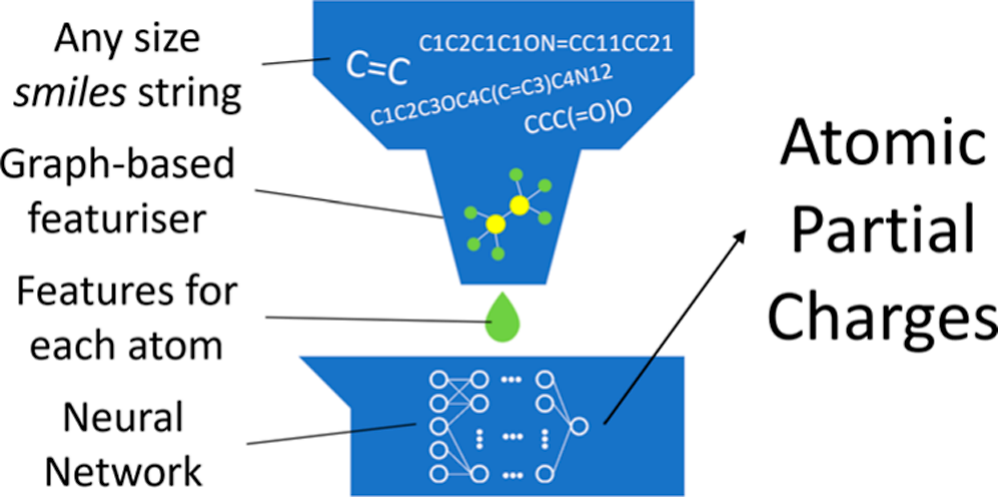

The atomic partial charge is of great importance in many fields, such as chemistry and drug-target recognition. However, conventional quantum-based computing of atomic charges is relatively slow, limiting further applications of atomic charge analysis. With the help of machine learning methods, various kinds of models appear to speed up atomic charge calculations. However, there are still some concerning problems. Some models based on geometric coordinates require high-accuracy geometry optimization as a preprocess, while other models have a limitation on the size of input molecules that narrow the applications of the model. Here, we propose a machine learning atomic charge model based on a message-passing featurizer. This preprocessing featurizer can quickly extract atomic environment information from a molecule according to the connectivity inside the molecule. The resulting descriptor can be used with a neural network to quickly predict the atomic partial charge. The model is able to automatically adapt to any size of molecule while remaining efficient and achieves a root-mean-square error in the Hirshfeld charge prediction of 0.018e, with an overall time complexity of *O*(*n*^2^). Thus, this model could enlarge the range of applications of atomic partial charge to more fields and cases.

## Introduction

The atomic partial charge is a widely used concept in computational chemistry. It is important in fields such as molecular properties analysis and drug-target recognition.^1,2^ However, the direct observation of atomic partial charge is not possible because it is not uniquely defined; hence, multiple definitions of computed charge distribution in molecules are widely accepted and used.^3^ The partial charges calculated by high-level quantum mechanics-based methods are relatively accurate but computationally expensive and time-consuming, compared to conventional semiempirical algorithms which are fast but with greater error.^4^ As the design of drug molecules continues to develop, there is a great demand of highly efficient methods to search and filter among millions of candidate molecules for proper properties like atomic charge distribution.^5^ Machine learning (ML) models that can scale to these large systems while maintaining sufficient accuracy are thus required. We propose a possible model here.

With the development of ML, recent research has started to focus on balancing time efficiency and accuracy in atomic partial charge prediction using ML algorithms. For example, Bleiziffer et al.^6^ proposed a random forest regression model in 2018 using atom-centered atomic pair fingerprints on DDEC6 charge^7^ and achieved a root-mean-square error (RMSE) of 0.016e on the testing set. Another approach is taken by PhysNet,^8^ which uses fully connected neural networks to predict energies, forces, and partial charges for molecular dynamics. A third method is used by Wang et al.^1^ who employ a message-passing neural network to build a model on 12 features of molecules and predicted DDEC6 charge with an RMSE of 0.0162e. In 2022, Gallegos created a model^2^ that focuses on the charge given by the quantum theory of atoms in molecules and is based on high-dimensional neural networks. The RMSE depends on the atom type, ranging from 0.0090 for H to 0.0221 for N.

Although many machine-learning-based atomic partial charge prediction models have been developed, there are several concerns that still need to be satisfied. Some models use the Cartesian coordinates as part of input features which requires a preprocess of geometry optimization. Another concern of most current models is the restriction on the size of molecules they can describe. In other words, once the models are trained, they can be applied only to other molecules with a maximum number of atoms defined by the training process.

Here, we introduce a coordinate-free and low-order scaling [*O*(*N*^2^) where *N* is the number of atoms in the molecule] ML model for quick atomic charge prediction for C, H, O, N, and potentially other elements, such as S and F. It is applicable to multiple kinds of organic molecules of any size, such as proteins and lead-like molecules. We propose a featurizer to aggregate and extract the local environment of atoms based on graph connectivity information. We use five key features for each atom inside a molecule to describe the atom itself and its surrounding environment. The normalized features are then passed to an artificial neural network (ANN) for atomic charge prediction. Our model can fit different kinds of atomic charge, such as Mulliken,^9^ Hirshfeld,^10^ semiempirical charge generated by AMSOL,^11^ CM5,^12^ and DDEC6.^7^

## Methods

### Message-Passing Featurizer

The idea of molecular graph convolutions started in 2016^13^ and message-passing neural networks (MPNN) were first proposed by Gilmer et al. in 2017^14^ to solve quantum chemistry problems based on graph neural networks. The original formulas of MPNN are as follows. The message function is defined by
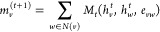
1while the update function is given by
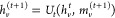
2and the readout function is
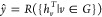
3Here, *m* is the message, *w* is a neighboring node (atom) of node *v*, *h* stands for the feature of a node (atom), *e* represents the feature of an edge (bond/connection), and *t* declares the current stage of the update. The quantities and *G* stand for the predicted result and the molecular graph. Finally, *M*_*t*_, *U*_*t*_, and *R* are the message function, update function, and readout function, respectively, where the learnable parameters are defined. Because many existing graph neural network (GNN) methods can be adapted into MPNNs, the mathematical definition of the message function, update function, and read-out function vary under different circumstances. Here, we only include the version based on “Gated GNN” (GG-NN)^15^ which is used by Gilmer. Further details can be found in the Supporting Information.

Under this circumstance, it is clear that the trainable parameters completely depend on the graph of a molecule, which restricts the size of the input graph. If the input molecule has a different size and shape from the training molecule, the model has to either generate ghost atoms (usually pad zeros) or predict results using insufficiently trained parameters. Worse still, the message generation and passing process are performed in every training epoch, which may significantly slow the training process.

In order to obtain the advantages and avoid the disadvantages of MPNN, we decided to move this message-passing algorithm into the preprocessing stage. By properly designing the message function and the update function, the number of hyper-parameters can be significantly reduced. This so-called message-passing featurizer (MPF) operates on the molecular graph and, for each atom, gives out extracted features that contain both the atomic properties and environmental information. These features are then passed to a dense neural network to predict the partial charge of this atom. Moreover, by using MPF, the neural network needs to predict the charge for only one atom each time. This allows the model to dynamically adapt to different sizes of molecules without the need for retraining and padding of zeros.

### Graph Implementation

In the MPF-powered model, each molecule is mapped to a graph space according to its atomic species and molecular connectivity. Atoms in the molecule are represented as nodes in the graph. Each atom has associated with it one unchangeable property, namely, its Pauling electronegativity. It is noted that the electronegativity does not provide any physical or chemical information but is used to specify the atomic type. As Pauling electronegativities take a narrow range of values, they make normalization of the descriptor easy. Nodes can be described by a vector, named the atom vector, containing the electronegativity of each atom in the molecule. For a molecule with *n* atoms, the atom vector is defined as
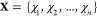
4where χ_*i*_ here denotes the electronegativity for atom *i* in the molecule.

The edges in the graph are the bonds in the molecule, which can be written in a 2-D matrix called the topology matrix **T**. The elements in **T** reads
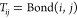
5Here, Bond(*i*, *j*) converts the bonding type of the bond between atom *i* and *j* into an integer and is defined by
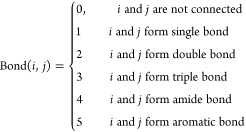
6For computational convenience, the adjacency matrix **A** is formed to describe the connectivity of the graph only, which is defined as
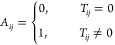
7

### Graph-Based Message Passing

The message-passing process in MPF aims to aggregate information from connected atoms and update the feature on every atom. After several rounds of updates, the feature of atoms can be regarded as a message and passed to further atoms and can help describe the local environment of the selected atom within a cutoff defined by the number of bonds traversed.

In this model, we define an update function for atom *i* for stage *l* + 1 as
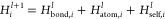
8Here, the updated feature *H*_*i*_ consists of a bond contribution term *H*_bond,*i*_, an atom contribution term *H*_atom,*i*_, and a self-contribution term *H*_self,*i*_. The bond contribution term reads
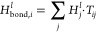
9The atomic contribution is defined as
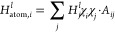
10and the self-contribution is
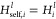
11Here, *j* denotes any atom inside the molecule.

In this model, the initial features for all atoms are set to 1, that is, , which gives the same weights for all atoms. Thus, the following updates can generate features in an unbiased way. The cutoff here is one of the most important hyper-parameters, which balances the accuracy with time and data efficiency. By increasing the cutoff value, the accuracy increases due to the consideration of longer-range interactions, but the required number of training data and the preprocessing time also increases. In this paper, all of these trained models use a cutoff parameter of 3, according to the size of the data set and the availability of computational resources.

### Atomic Featurization

Five features are used to describe the atomic local environment for atom *i*. Apart from the bond contribution , the atom contribution , and the self-contribution  based on the updated graph, two additional features, mean neighboring electronegativity *H*_MNE,*i*_ and self-electronegativity *H*_SEN,*i*_, are added. These can be written as
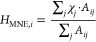
12and
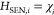
13Statistically, these five features are regarded as labels that describe the local atomic environment and which enable the following ANN to classify each atom and allocate the same partial charge to those atoms with the same labels.

Each of these five features is then normalized by its standard deviation, where the normalized feature  is defined by
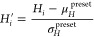
14Here,  and  denote the average value and standard deviation of feature *H* over the training data set and are computed after the updates. The final feature matrix **F**_*i*_ for atom *i* is then
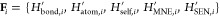
15The MPF shall be now fully defined.

### Neural Network Implementation

**Figure 1:**
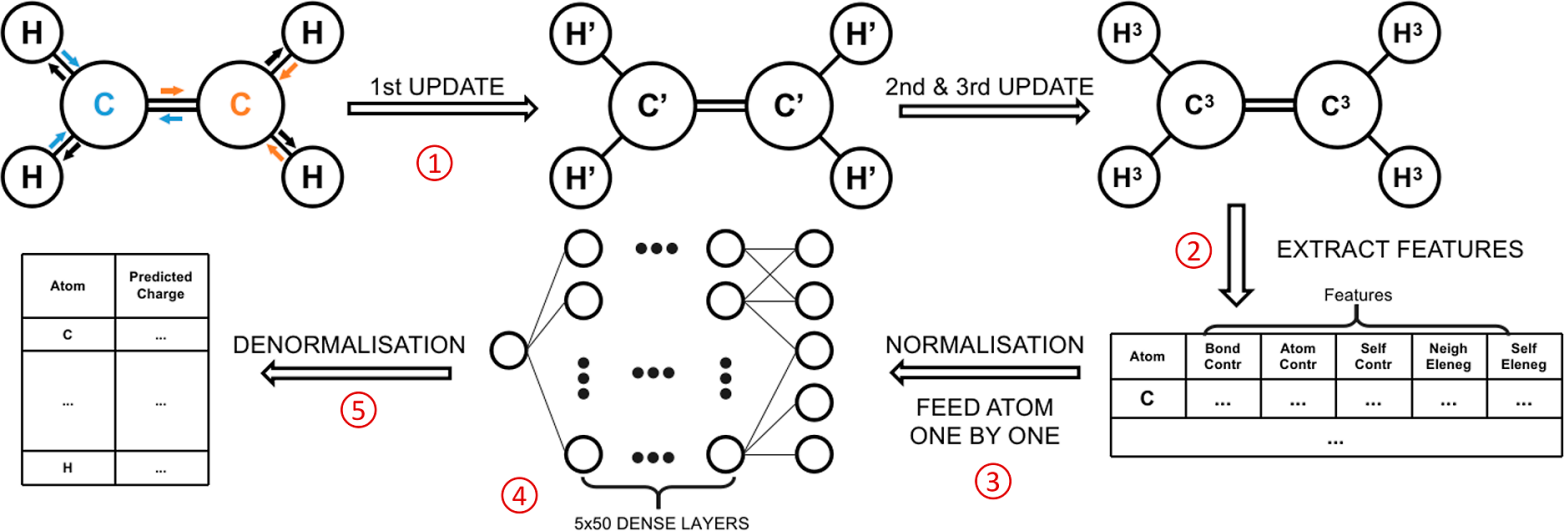
This figure summarizes the general structure of an MPF-based model. The model contains five key stages which are updates, featurization, normalization, ML-processing, and denormalization. In stage 1, the model will update every atom inside the molecule according to its topological information. For each atom, these updates will embed the features and properties of its nearest neighbors in the graph into a new feature. After several rounds of the update process, the featurizer in stage 2 will process these updated features and extract five key features for each atom. In stage 3, these extracted features will be normalized and then passed to the machine-learning model in stage 4. The output from the ML model will then be denormalized into the final result in stage 5.

Here, we use TensorFlow 2.7.4^16^ as the framework to implement our neural network. The general structure of our algorithm is listed in Figure 1. The model contains a 5-node input layer, five 50-node dense layers activated by the tanh function, and a 1-node output layer. This model accepts feature matrix **F**_*i*_ as input and returns a normalized predicted charge . Then,  is denormalized into the final predicted charge *Q*_*i*_ using the following postprocessing formula
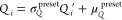
16where  and  are the standard deviation and average value of the final predicted charge *Q*, respectively, in the training data set.

This neural network model is trained for 100 epochs with the Adam optimizer^17^ (lr = 0.001, β_1_ = 0.9, β_2_ = 0.999, and ε = 10^–7^). The loss function is the mean absolute error (MAE) and the batch size is 16 for “Model Mulliken”, “Model Hirshfeld”, “Model CM5” and “Model DDEC6”, and 768 for “Model SE”. The training details can be found in the Supporting Information.

### Input Data Set

We use five data sets to train and validate our model for various charge types: “Model Mulliken”, “Model Hirshfeld”, “Model SE”, “Model CM5”, and “Model DDEC6”. Here, we use “Model Mulliken”, “Model Hirshfeld”, “Model CM5”, and “Model DDEC6” to demonstrate the flexibility and robustness of our model under multiple definitions of charge. “Model SE” is used to validate the performance of the model in a larger data set.

The molecular structures used in “Model Mulliken”, “Model Hirshfeld”, “Model CM5”, and “Model DDEC6” are obtained from database **GDB13**(18) with C, N, O, and H elements in the smiles format, with a total of 12,124 molecules in the training set and 1393 molecules in the testing set. Note that the smiles format gives the connectivity between atoms within a molecule but not the positions of the atoms. The format conversion from smiles to *xyz* (which does provide atomic positions) is done by **Open Babel**.^19^ Here, a conformational search and optimization method^20^ is used. When converting a smiles string to coordinates, this method seeks to find the conformation that has the lowest computational energy. We note that this means that the model can only be used reliably for minimum energy conformations. All of the Mulliken, Hirshfeld, and CM5 charges are calculated by **Gaussian 16**(21) using the B3LYP functional and a 6-31G(d) basis set. In this DFT section, we optimized all these molecules to their lowest energy state. In “Model DDEC6”, the charge is obtained via program **Chargemol**(7,22) based on the electron density provided by **Gaussian 16**. In “Model SE”, both the training and testing data sets are obtained from the **ZINC20** database in the mol2 format with **AMSOL**-calculated semiempirical charges.^23^ A total of 898,466 lead-like molecules are selected for training and another 100,000 lead-like molecules for testing. The selected data set and the detailed information on the four data sets are shown in the Supporting Information.

For the testing and predicting processes, the accepted input formats are smiles and mol2. For smiles input, the string is first converted to mol2 format by Open Babel without generating atomic coordinates.^19^

## Results and Discussion

### Accuracy Analysis

**Figure 2:**
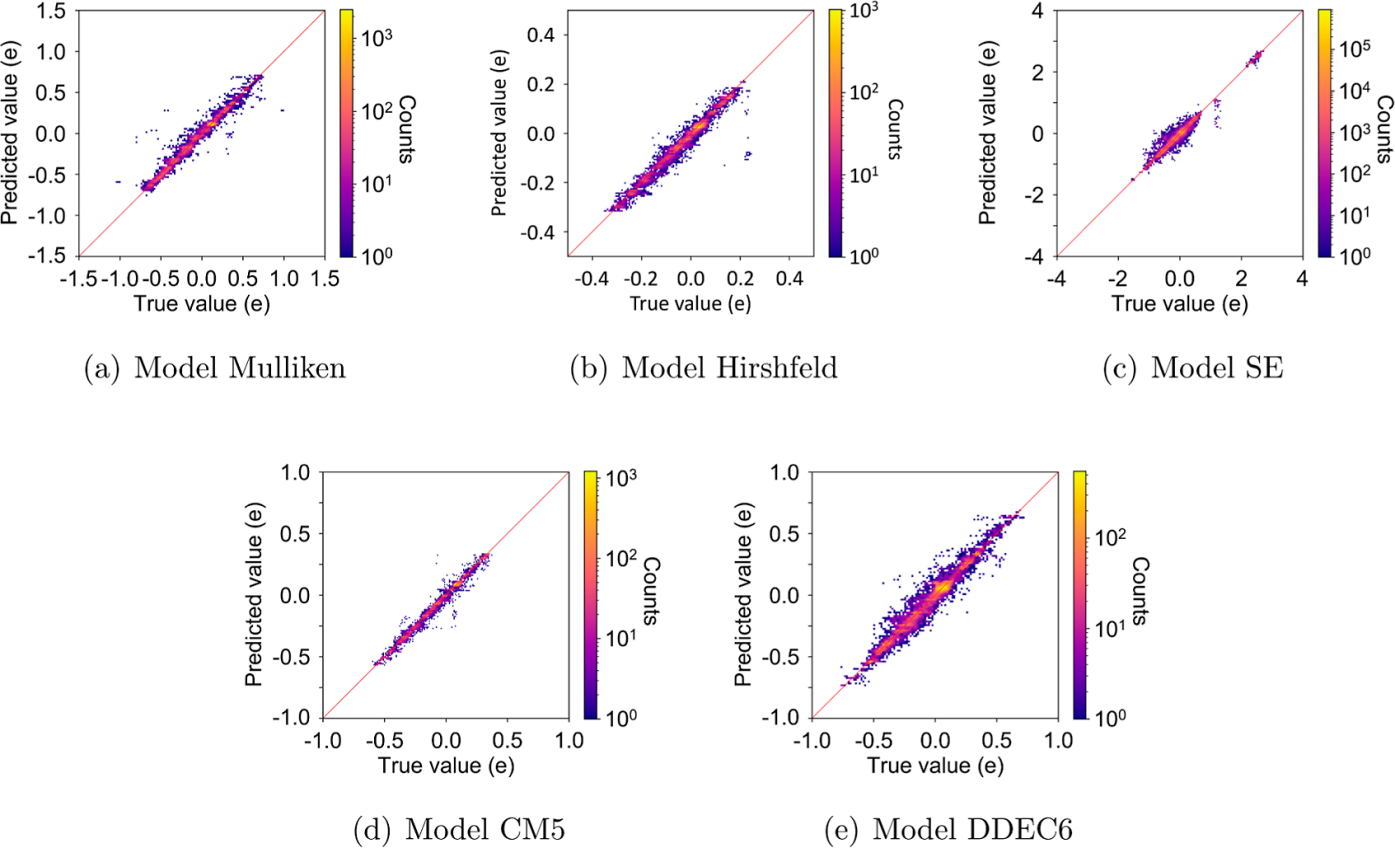
Heatmaps of the predicted value of atomic charge in units of the electron charge *e* against the true value for four models. Note the variation in scales between models.

We use seven key indicators to evaluate the accuracy of these models: mean absolute error, median absolute error, the highest absolute error from the lowest 80% of errors [which we call the top low 80% absolute error (TL80AE)], median absolute percentage error, the highest absolute percentage error from the lowest 80% of errors [which we call the top low 80% percentage error (TL80PE)], coefficient of determination (*R*^2^), and RMSE. Table 2 shows the value of indicators for each model and element, and Figure 2 shows the heatmap of predicted value against the true value for the testing set for each model. Due to the different definitions in each charge model, the value of the assigned charge on an atom is on a different scale for each model. So the general comparison below only focuses on the median of absolute percentage error, the absolute percentage error of 80% prediction, and *R*^2^.

**Table 1:** Detailed Information for the Selected Datasets

	ZINC training	ZINC testing	GDB13 training	GDB13 testing
charge type	semiempirical		Mulliken/Hirshfeld/CM5/DDEC6	
number of molecules	898,466	100,000	12,124	1393
largest number of atoms	52	51	23	21
total number of atoms	35,532,841	3,907,949	203,052	21,044
number of C atoms	10,998,813	1,228,545	61,980	6858
number of H atoms	17,828,785	1,954,366	108,043	9900
number of O atoms	2,782,219	344,935	15,576	2116
number of N atoms	3,690,163	354,572	17,453	2170
number of F atoms	74,996	11,705	N/A	N/A
number of S atoms	152,431	23,033	N/A	N/A
number of Cl atoms	4955	716	N/A	N/A
number of Br atoms	344	77	N/A	N/A

**Table 2:** Accuracy Analysis on the Testing Data Set by Elements. When Data Is Not Available, the Entry Is Marked as “N/A”

indicator	model	all	C	H	O	N	F	S	Cl	Br
mean absolute error (e)	Mulliken	0.023	0.035	0.015	0.020	0.028	N/A	N/A	N/A	N/A
	Hirshfeld	0.011	0.011	0.008	0.014	0.017	N/A	N/A	N/A	N/A
	SE	0.014	0.018	0.011	0.011	0.014	0.010	0.032	0.022	0.045
	CM5	0.012	0.014	0.008	0.016	0.020	N/A	N/A	N/A	N/A
	DDEC6	0.029	0.042	0.021	0.020	0.031	N/A	N/A	N/A	N/A
median absolute error (e)	Mulliken	0.015	0.022	0.011	0.015	0.018	N/A	N/A	N/A	N/A
	Hirshfeld	0.007	0.007	0.007	0.009	0.010	N/A	N/A	N/A	N/A
	SE	0.008	0.009	0.008	0.007	0.007	0.008	0.013	0.017	0.038
	CM5	0.008	0.008	0.006	0.010	0.010	N/A	N/A	N/A	N/A
	DDEC6	0.018	0.027	0.014	0.011	0.018	N/A	N/A	N/A	N/A
TL80AE (e)	Mulliken	0.034	0.053	0.025	0.032	0.040	N/A	N/A	N/A	N/A
	Hirshfeld	0.016	0.017	0.013	0.021	0.024	N/A	N/A	N/A	N/A
	SE	0.020	0.026	0.018	0.016	0.020	0.015	0.042	0.033	0.069
	CM5	0.017	0.021	0.013	0.024	0.028	N/A	N/A	N/A	N/A
	DDEC6	0.044	0.067	0.035	0.029	0.045	N/A	N/A	N/A	N/A
median absolute percentage error	Mulliken	7.039%	13.468%	6.776%	3.027%	5.011%	N/A	N/A	N/A	N/A
	Hirshfeld	12.423%	18.756%	13.129%	4.516%	8.767%	N/A	N/A	N/A	N/A
	SE	5.166%	6.758%	8.318%	1.436%	1.346%	4.414%	0.753%	57.640%	77.422%
	CM5	5.781%	10.613%	5.252%	3.195%	2.971%	N/A	N/A	N/A	N/A
	DDEC6	12.789%	22.288%	14.291%	2.932%	6.424%	N/A	N/A	N/A	N/A
TL80PE	Mulliken	19.505%	44.977%	15.979%	7.336%	11.717%	N/A	N/A	N/A	N/A
	Hirshfeld	30.681%	64.528%	26.100%	10.686%	29.120%	N/A	N/A	N/A	N/A
	SE	18.709%	25.509%	19.661%	3.395%	4.592%	9.513%	4.096%	391.451%	293.597%
	CM5	15.795%	34.391%	12.157%	8.976%	9.333%	N/A	N/A	N/A	N/A
	DDEC6	43.384%	81.166%	39.288%	8.351%	21.028%	N/A	N/A	N/A	N/A
RMSE (e)	Mulliken	0.040	0.058	0.021	0.026	0.051	N/A	N/A	N/A	N/A
	Hirshfeld	0.018	0.017	0.010	0.019	0.036	N/A	N/A	N/A	N/A
	SE	0.026	0.036	0.017	0.022	0.027	0.015	0.070	0.032	0.057
	CM5	0.020	0.022	0.011	0.023	0.038	N/A	N/A	N/A	N/A
	DDEC6	0.045	0.061	0.030	0.033	0.052	N/A	N/A	N/A	N/A
*R*^2^	Mulliken	0.981	0.956	0.942	0.907	0.913	N/A	N/A	N/A	N/A
	Hirshfeld	0.971	0.941	0.939	0.874	0.785	N/A	N/A	N/A	N/A
	SE	0.994	0.978	0.968	0.984	0.983	0.750	0.995	0.399	–0.366
	CM5	0.990	0.968	0.984	0.937	0.892	N/A	N/A	N/A	N/A
	DDEC6	0.965	0.926	0.911	0.905	0.918	N/A	N/A	N/A	N/A

By comparing these indicators and heatmaps for each model, we discover that our algorithm can predict the various kinds of charges with a value of *R*^2^ larger than 0.96 and a narrow distribution on heatmaps. By using the RMSE as the primary indicator for accuracy analysis, it can be concluded that “Model Hirshfeld” and “Model CM5” are the two models of best accuracy. However, it can be found that the result of the accuracy analysis can be different when based on different indicators. One possible reason for such an observation is the difference in the absolute values of different types of charges and the sensitivity of an indicator to different aspects. By comparing Figure 2a,b, the distribution range of the absolute value of the Mulliken charge is greater than that of the Hirshfeld charge. So in Table 2, although “Model Hirshfeld” has a lower value of MAE and TL80AE compared to “Model Mulliken”, it has a greater value in MAPE, TL80PE, and R^2^. Thus, these indicators need to be interpreted with a reference to the aspects to which they are sensitive to.

By comparing the results for each element, our model manages to predict charges on C, H, O, N, F, and S atoms with *R*^2^ greater than 0.75. More specifically, these trained models achieve better accuracy on elements O, N, F, and S. The accuracy of these models depends on the amount of data available in the training set and the distribution range of the charges. For instance, Cl and Br have worse accuracy than other elements due to the limited data availability in the training set, as seen in Table 1. In addition, for the training set of “Model Hirshfeld” for example, the charge distribution of C (−0.185 to 0.237) is wider than that of H (0.007 to 0.205), and the RMSE value of C is greater than that of H, as shown in Table 1.

Although the MPF is designed for quick filtering and the flexibility to perform calculations for any size of molecule, the MPF-based model still provides acceptable accuracy when compared with other existing DDEC charge predicting methods. The MPF-based model for DDEC6 charge prediction introduced has an overall RMSE of 0.045e in the GDB13-based testing set. Bleiziffer’s models^6^ achieved RMSEs of 0.029e and 0.016e in a testing set based on ZINC and ChEMBL databases. The model DeepAtomicCharge^1^ has an RMSE of 0.0162e in their ZINC- and ChEMBL-based databases.

### Time Complexity Analysis

**Figure 3:**
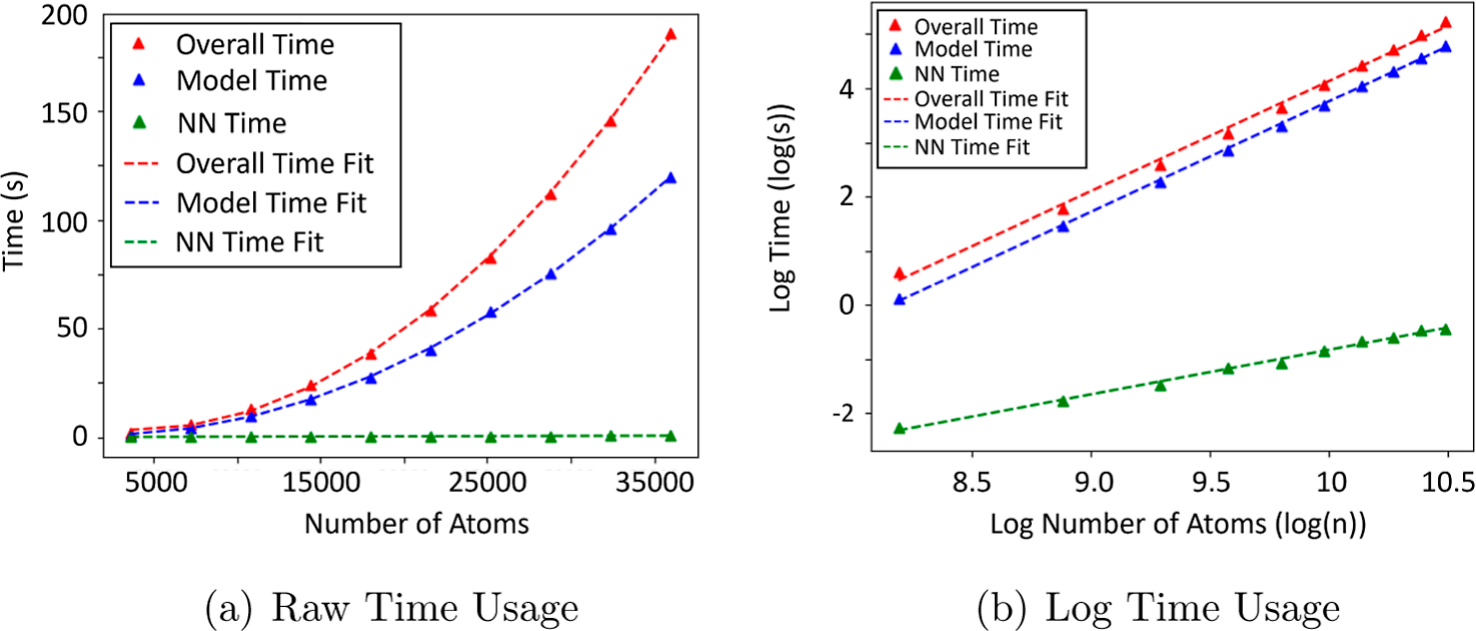
Time complexity test on “Model SE”. Panel (a) shows the raw time usage against the number of atoms. The dashed curves are second-order polynomials fitted to the data. Panel (b) shows the log of the time usage against the log of the number of atoms. The dashed lines are first-order polynomials fitted to the data.

The charges are computed one atom at a time, so the time used by the neural network to predict the charges on all of the atoms in a molecule increases linearly as the number of atoms increases. However, the format conversion from smiles to mol2 and data preprocessing, i.e., the updating process on the graph and feature extraction, are more time-consuming. Figure 3 shows the results of the time efficiency test on 10 alkane chains containing from 1200 to 12,000 carbon atoms. Each test was run five times, and the average time usage was taken. The overall time test measures the time starting from reading the smiles string, including the time used to convert it to the mol2 format via Open Babel, mol2 file read-in, data preprocessing, and the neural network prediction. The model time test consists only of the time for data preprocessing and neural network prediction. And the NN Time only measures the time that neural network prediction used. These results indicate the overall time complexity of the MPF algorithm is *O*(*n*^2^) and that of the neural network prediction is linear (*O*(*n*)). Detailed information on the tested points and results and the specifications for the machine used to perform the tests is given in the Supporting Information.

## Conclusions

In this paper, a new machine-learning featurizer named MPF for atomic partial charge prediction is presented. The MPF-based model has several advantages:

- It is totally coordinate-free: this model accepts a smiles string as input and does not require Cartesian coordinates.
- It is molecule size independent: our model makes charge predictions for any size of molecule without any retraining and modification of the existing model.
- It is fast and accurate: the time complexity of the MPF-based model is *O*(*n*^2^). The median absolute percentage error of our models is in the range 5 to 12.5%, the exact value depending on the type of partial charge.

It is important to clarify that our MPF algorithm still has some limitations. First, in the current work, we only considered up to the third nearest neighbor of each atom, which means our model ignores long-range interactions. Second, the bond length, bond angle, and torsion angle are not taken into consideration in our model. So this model may fail to predict accurate partial charges in the situation that secondary structure matters. Third, this model is designed for single, double, triple, amide, and aromatic bonds only and may fail in cases where H bonds and polarization are important. Another limitation of this work is that it is only reliable for the lowest energy conformations as the training set only contained lowest energy conformations. Some of these limitations might be overcome through the use of larger cutoffs and broader training sets. In order to describe high energy conformations, the input smiles string would need to be augmented with additional information, and the training set would need to be extended to include high energy conformations.

## Data Availability

**Gaussian 16** is used for DFT calculation in “Model Mulliken”, “Model Hirshfeld”, “Model CM5”, and “Model DDEC6”.^21^**Open Babel** is used for data format conversions, including from *smiles* to mol2 and from Gaussian’s log to mol2.^19^**Chargemol** is the program used for DDEC6 charge calculation in “Model DDEC6”, which can be accessed via https://github.com/berquist/chargemol.^7,22^**cppgd** is used for Gaussian’s log files and mol2 files read in, which can be accessed via https://github.com/xieqin74123/cppgd. All training and testing data sets and Python scripts are provided in 10.5281/zenodo.10149110.

## Supporting Information Available

Training and testing data sets extracted from ZINC and GDB13, definitions of GG-NN-based MPNN model, training and testing detail information for “Model Mulliken”, “Model Hirshfeld”, “Model SE”, “Model CM5”, and “Model DDEC6”, raw data and machine specification of time complexity test, and factorial experiment raw data and analysis (PDF)

## References

[ref1] Wang J.; Cao D.; Tang C.; Xu L.; He Q.; Yang B.; Chen X.; Sun H.; Hou T. DeepAtomicCharge: a New Graph Convolutional Network-based Architecture for Accurate Prediction of Atomic Charges. Briefings Bioinf. 2021, 22, bbaa183 10.1093/bib/bbaa183. 34020543

[ref2] Gallegos M.; Guevara-Vela J. M.; Pendás Á. M. NNAIMQ: A Neural Network Model for Predicting QTAIM Charges. J. Chem. Phys. 2022, 156, 014112 10.1063/5.0076896. 34998318

[ref3] Zhao D.-X.; Zhao J.; Zhu Z.-W.; Zhang C.; Yang Z.-Z. A Model of Atoms in Molecules Based on Potential Acting on One Electron in a Molecule: I. Partition and Atomic Charges Obtained from Ab Initio Calculations. Int. J. Quantum Chem. 2018, 118, e25610 10.1002/qua.25610.

[ref4] Xu L.; Sun H.; Li Y.; Wang J.; Hou T. Assessing the Performance of MM/PBSA and MM/GBSA Methods. 3. The Impact of Force Fields and Ligand Charge Models. J. Phys. Chem. B 2013, 117, 8408–8421. 10.1021/jp404160y. 23789789

[ref5] Wu K.; Karapetyan E.; Schloss J.; Vadgama J.; Wu Y. Advancements in Small Molecule Drug Design: A Structural Perspective. Drug Discovery Today 2023, 28, 103730 10.1016/j.drudis.2023.103730. 37536390 PMC10543554

[ref6] Bleiziffer P.; Schaller K.; Riniker S. Machine Learning of Partial Charges Derived from High-quality Quantum-mechanical Calculations. J. Chem. Inf. Model. 2018, 58, 579–590. 10.1021/acs.jcim.7b00663. 29461814

[ref7] Manz T. A.; Limas N. G. Introducing DDEC6 Atomic Population Analysis: part 1. Charge Partitioning Theory and Methodology. RSC Adv. 2016, 6, 47771–47801. 10.1039/C6RA04656H. PMC9096813 35703680

[ref8] Unke O. T.; Meuwly M. PhysNet: A Neural Network for Predicting Energies, Forces, Dipole Moments, and Partial Charges. J. Chem. Theory Comput. 2019, 15, 3678–3693. 10.1021/acs.jctc.9b00181. 31042390

[ref9] Mulliken R. S. Electronic Population Analysis on LCAO–MO Molecular Wave Functions. I. J. Chem. Phys. 1955, 23, 1833–1840. 10.1063/1.1740588.

[ref10] Hirshfeld F. L. Bonded-atom Fragments for Describing Molecular Charge Densities. Theor. Chim. Acta 1977, 44, 129–138. 10.1007/BF00549096.

[ref11] Cramer C. J.; Truhlar D. G. AM1-SM2 and PM3-SM3 Parameterized SCF Solvation Models for Free Energies in Aqueous Solution. J. Comput.-Aided Mol. Des. 1992, 6, 629–666. 10.1007/BF00126219. 1291630

[ref12] Marenich A. V.; Jerome S. V.; Cramer C. J.; Truhlar D. G. Charge Model 5: An Extension of Hirshfeld Population Analysis for the Accurate Description of Molecular Interactions in Gaseous and Condensed Phases. J. Chem. Theory Comput. 2012, 8, 527–541. 10.1021/ct200866d. 26596602

[ref13] Kearnes S.; McCloskey K.; Berndl M.; Pande V.; Riley P. Molecular Graph Convolutions: Moving Beyond Fingerprints. J. Comput.-Aided Mol. Des. 2016, 30, 595–608. 10.1007/s10822-016-9938-8. 27558503 PMC5028207

[ref14] Gilmer J.; Schoenholz S. S.; Riley P. F.; Vinyals O.; Dahl G. E. Neural Message Passing for Quantum Chemistry. In International Conference on Machine Learning; PMLR, 2017; pp 1263–1272.

[ref15] Li Y.; Tarlow D.; Brockschmidt M.; Zemel R. Gated Graph Sequence Neural Networks. arXiv preprint arXiv:1511.05493 2015.

[ref16] Abadi M.; TensorFlow: Large-Scale Machine Learning on Heterogeneous Systems. 2015, https://www.tensorflow.org/, Software available from tensorflow.org. (accessed 16 May 2024).

[ref17] Kingma D. P.; Ba J. Adam: A Method for Stochastic Optimization. 2014, arXiv:1412.6980. arXiv preprint. https://arxiv.org/abs/1412.6980.

[ref18] Blum L. C.; Reymond J.-L. 970 Million Druglike Small Molecules for Virtual Screening in the Chemical Universe Database GDB-13. J. Am. Chem. Soc. 2009, 131, 8732–8733. 10.1021/ja902302h. 19505099

[ref19] O’Boyle N. M.; Banck M.; James C. A.; Morley C.; Vandermeersch T.; Hutchison G. R. Open Babel: An Open Chemical Toolbox. J. Cheminf. 2011, 3, 33 10.1186/1758-2946-3-33. PMC3198950 21982300

[ref20] Yoshikawa N.; Hutchison G. R. Fast, Efficient Fragment-based Coordinate Generation for Open Babel. J. Cheminf. 2019, 11, 49 10.1186/s13321-019-0372-5. PMC6676618 31372768

[ref21] Frisch M. J.; Gaussian 16, revision C.01. 2016; Gaussian Inc. Wallingford CT.

[ref22] Limas N. G.; Manz T. A. Introducing DDEC6 Atomic Population Analysis: part 2. Computed Results for a Wide Range of Periodic and Nonperiodic Materials. RSC Adv. 2016, 6, 45727–45747. 10.1039/C6RA05507A.

[ref23] Irwin J. J.; Shoichet B. K. ZINC - a Free Database of Commercially Available Compounds for Virtual Screening. J. Chem. Inf. Model. 2005, 45, 177–182. 10.1021/ci049714+. 15667143 PMC1360656

