# Supplementary material for: Coordinate-Free and Low-Order Scaling Machine Learning Model for Atomic Partial Charge Prediction for Any Size of Molecules
Source: J Chem Inf Model. 2024 May 17;64(11):4419–25. doi: 10.1021/acs.jcim.4c00376 (PMC11167589; doi:10.1021/acs.jcim.4c00376)
Supplement: Supplementary file 1 — ci4c00376_si_001.pdf [file ci4c00376_si_001.pdf]

A coordinate-free and low order scaling machine learning model for atomic partial charge prediction for any size of molecules: Supporting Information

Qin Xie and Andrew P. Horsfield*

Department of Materials, Imperial College London, SW7 2AZ, London

E-mail: a.horsfield@imperial.ac.uk

Training and testing data set

The training data for "Model SE" that selected from ZINC database are: "CAAAMN.xaa", "CAAARN.xaa", "CAABMN.xaa", "CAABRN.xaa", "CAACRN.xaa", "CAADMN.xaa", "CAADRN.xaa", "CABAMN.xaa", "CABARN.xaa", "CABBRN.xaa", "CABDMN.xaa", "CABDRN.xaa", "CACAMN.xaa", "CACARN.xaa", "CACBMN.xaa", "CACBRN.xaa", "CACCRN.xaa", "CACDMN.xaa", "CACDRN.xaa", "CAEAMN.xaa", "CAEARN.xaa", "CAEBMN.xaa", "CAEBRN.xaa", "CAEDMN.xaa", "CAEDRN.xaa", "CBAAMN.xaa", "CBAARN.xaa", "CBABMN.xaa", "CBABRN.xaa", "CBADMN.xaa", "CBADMN.xab", "CBADMN.xac", "CBADMN.xad", "CBADMN.xae", "CBADRN.xaa" and "CBADRN.xab". The testing data for "Model SE" selected from ZINC database is "CBADRN.xac".

Please note that some of the molecules in these files may be corrupted or contain unsupported elements. These molecules will be automatically dropped by the read-in programme **cppgd**.

The GDB13 training data are collected from "GDB13-CNO" database, in file "4.cno.smi", "5.cno.smi", "6.cno.smi", "7.cno.smi" and "8.con.smi". The GDB13 testing data are from "8.cno.smi" only. Detailed information can be found in datasets provided.

GG-NN Based MPNN Model

The following summary is based on Gilmer's publication in 2020.[1] The message function used in GG-NN based models is:

$$M_t(h_v^t, h_w^t, e_{vw}) = A_{e_{vw}} h_w^t. \tag{1}$$

Here, $A_{e_{vw}}$ is learnable for edge label e_{vw}. The update function is

$$U_t = \mathrm{GRU}(h_v^t, m_v^{t+1}). \tag{2}$$

The GRU here is the Gated Recurrent Unit,[2] which is a kind of recurrent neural network. The GRU performs similarly to the "long-short term memory" (LSTM) method but is computationally cheaper, according to the Microsoft Research Asia's Natural Language Computing Group.[3] At each update stage t, the update function used is the same, and the readout function is

$$R = \sum_{v \in V} \sigma\left(i(h_v^T, h_v^0)\right) \odot \left(j(h_v^T)\right). \tag{3}$$

Here, $\odot$ is the Hadamard product, or element-wise product, of the matrices and i and j index the neural networks.

Model Detail

“Model Mulliken”

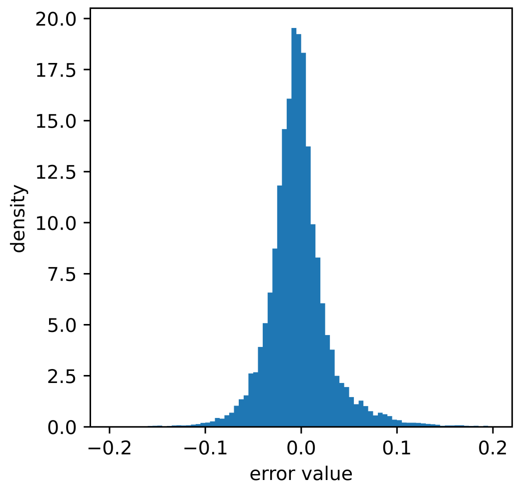

(a) Histogram of errors

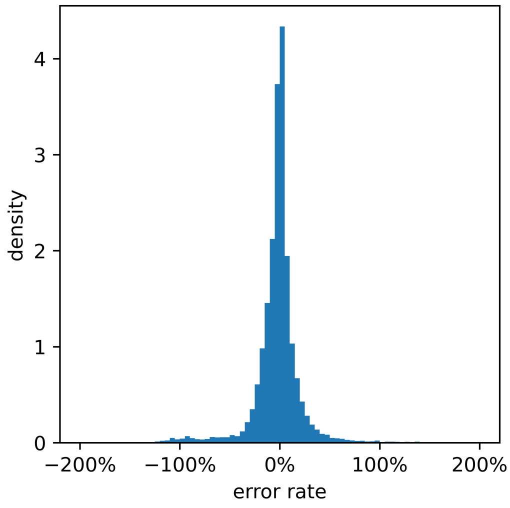

(b) Histogram of percentage errors

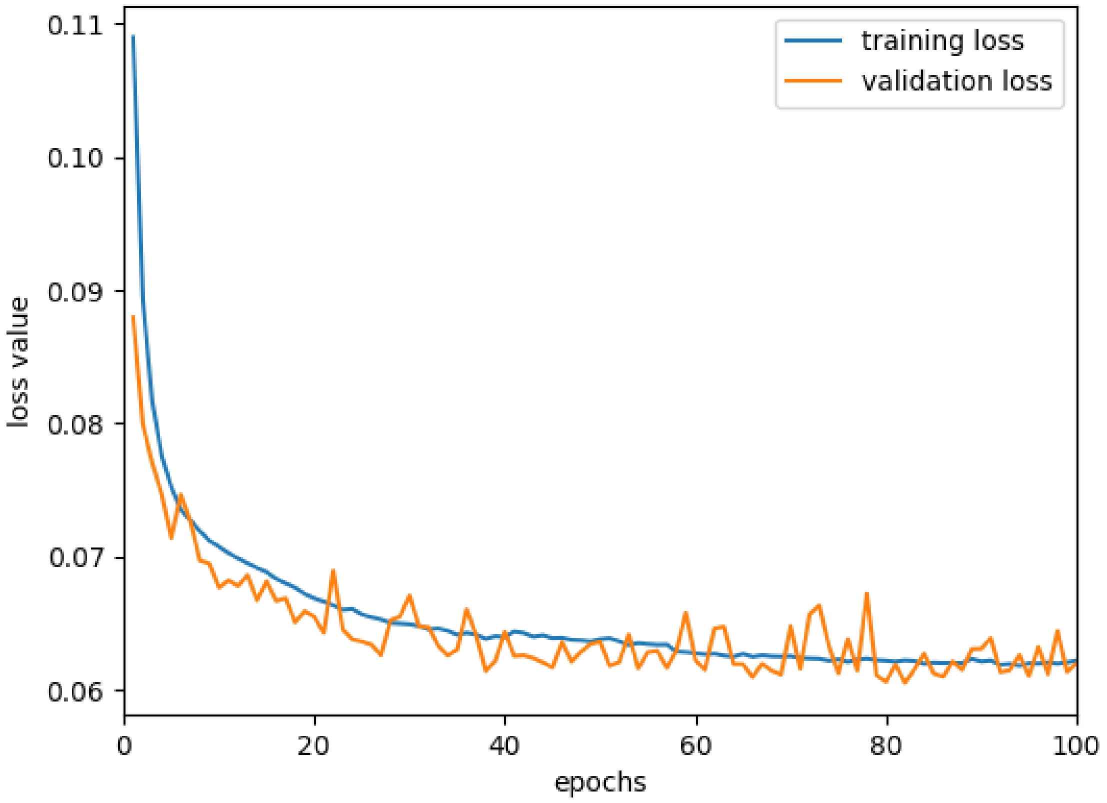

(c) Training loss in epochs

“Model Hirshfeld”

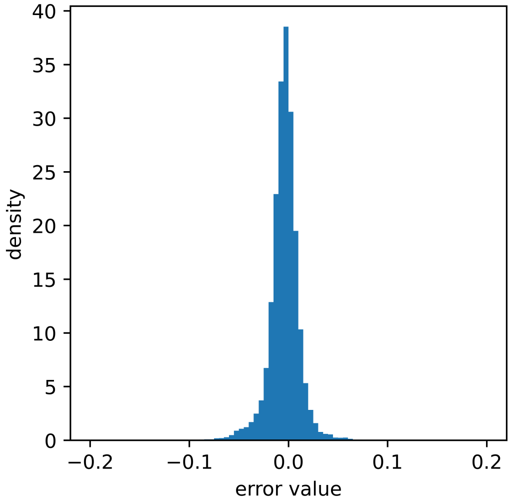

(d) Histogram of errors

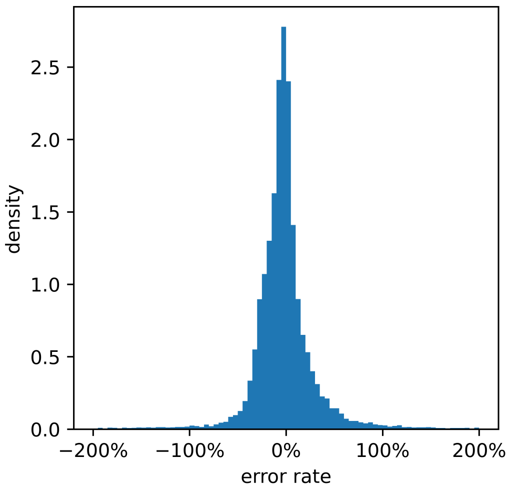

(e) Histogram of percentage errors

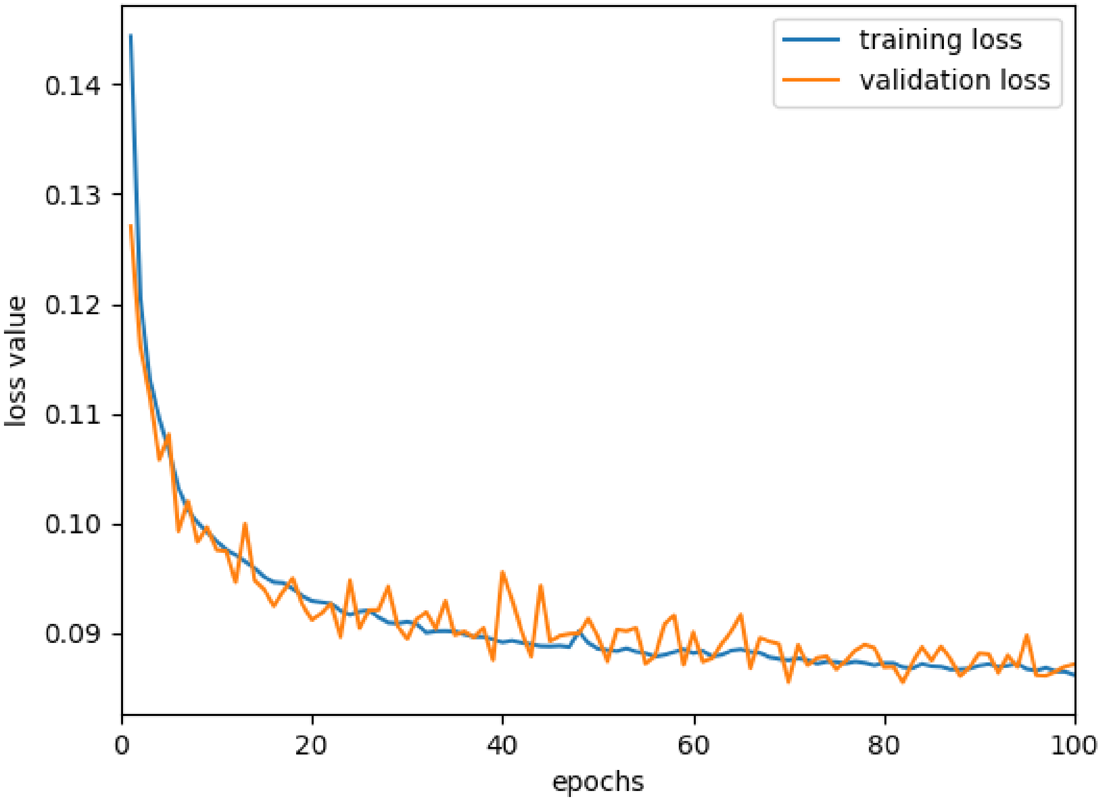

(f) Training loss in epochs

“Model SE”

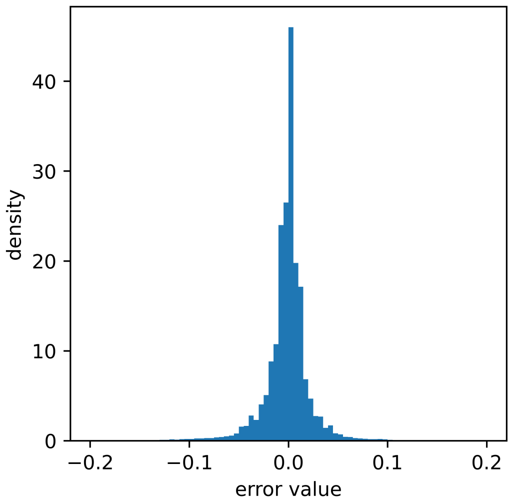

(g) Histogram of errors

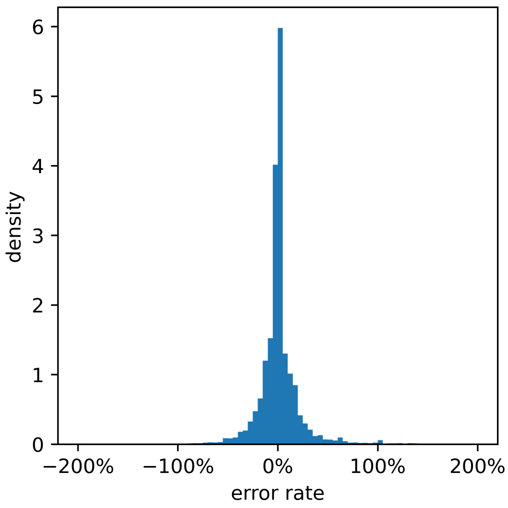

(h) Histogram of percentage errors

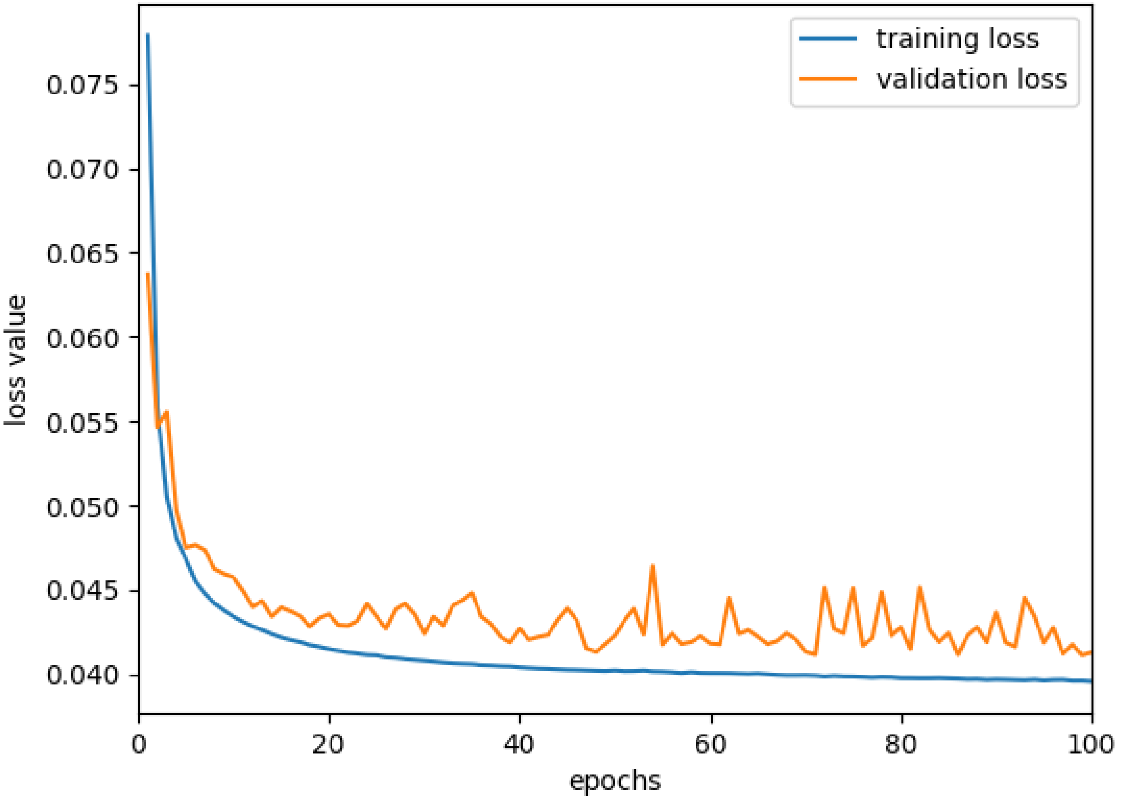

(i) Training loss in epochs

“Model CM5”

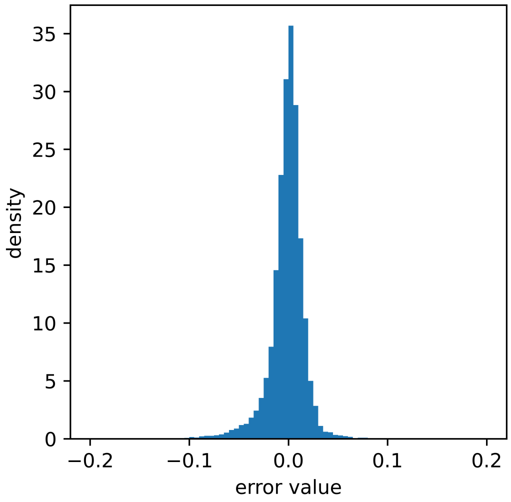

(j) Histogram of errors

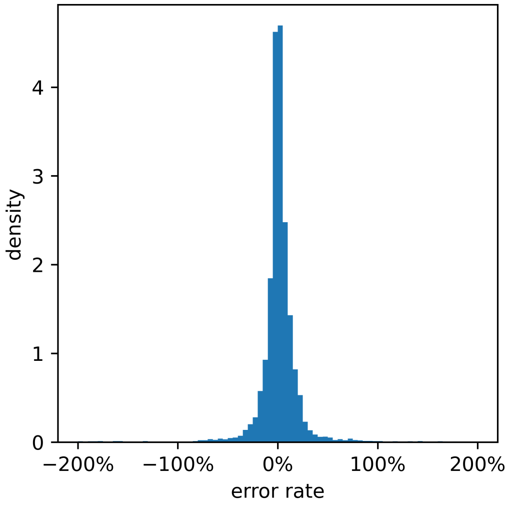

(k) Histogram of percentage errors

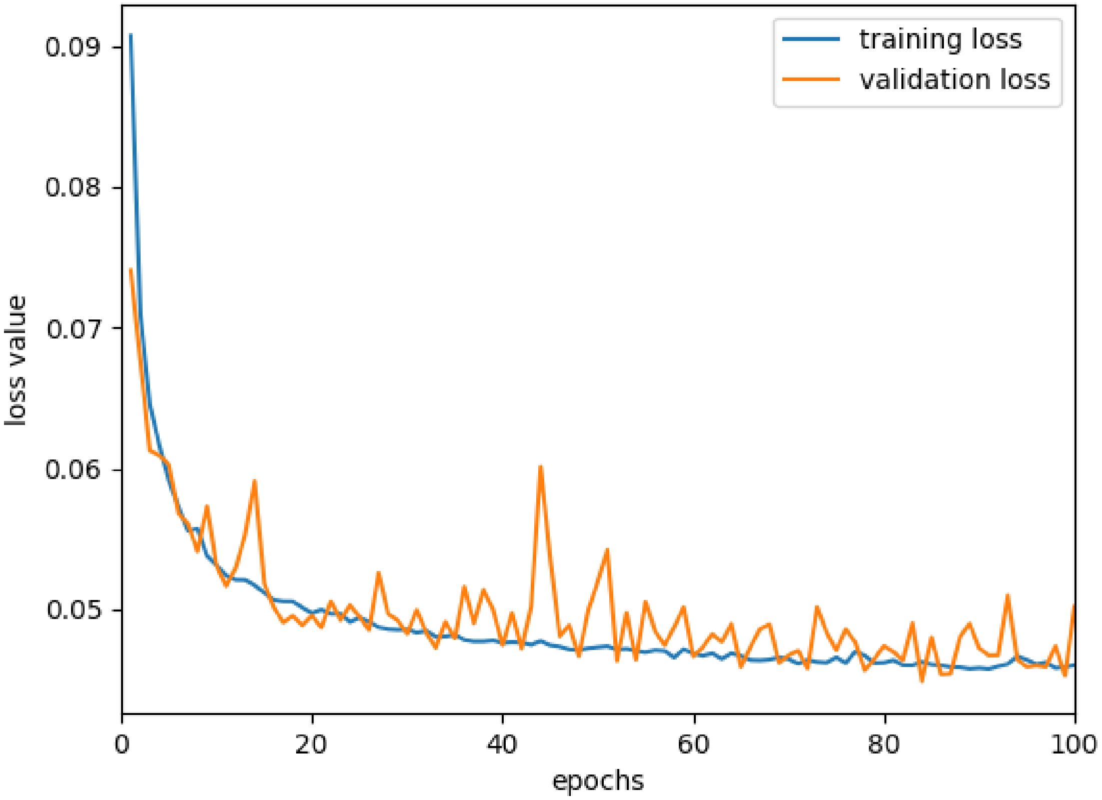

(l) Training loss in epochs

“Model DDEC6”

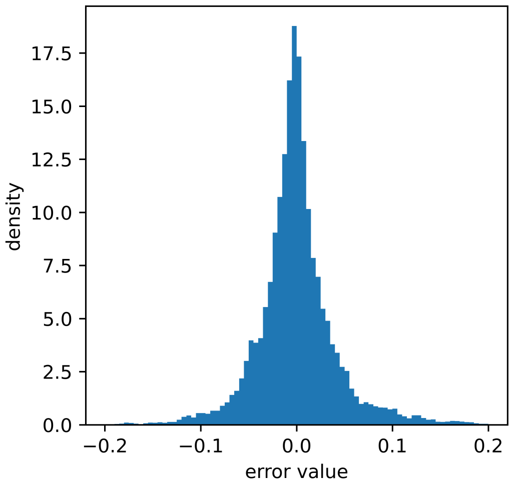

(m) Histogram of errors

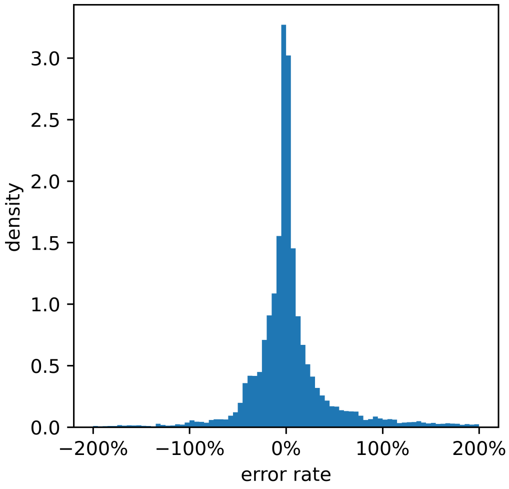

(n) Histogram of percentage errors

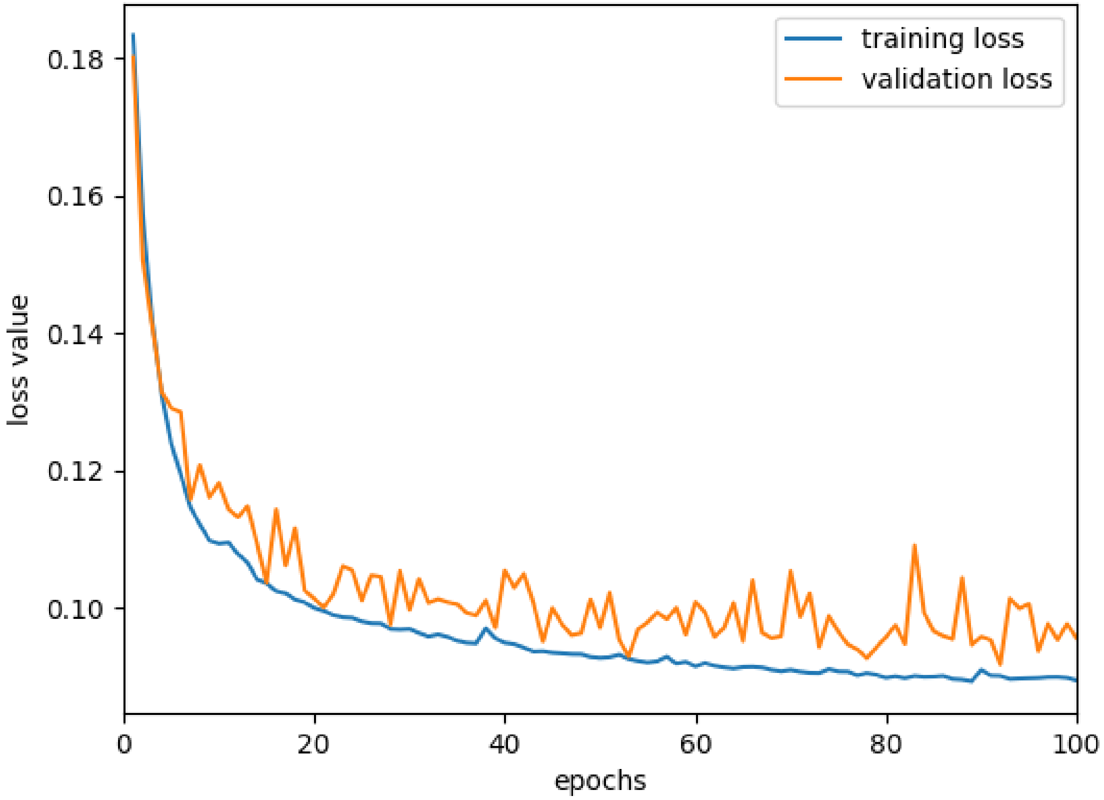

(o) Training loss in epochs

Time Complexity Test Detail

Table S1: time complexity test detail

Carbon Number	Atom Number	Overall Time (s)	Model Time (s)	NN Time (s)
1200	3602	1.86	1.12	0.10
2400	7202	5.94	4.36	0.17
3600	10802	13.28	9.67	0.23
4800	14402	24.03	17.35	0.31
6000	18002	38.29	27.29	0.34
7200	21602	58.24	40.26	0.43
8400	25202	82.89	57.67	0.51
9600	28802	112.15	75.34	0.54
10800	32402	145.57	95.89	0.63
12000	36002	185.70	119.67	0.63

Tested machine specifications:

CPU: Intel Xeon E2286M 8c16t @5.0GHz

GPU: Nvidia Quadro RTX 3000 Mobile

Memory: 64 GB DDR4 2667MHz

OS: Deepin 20.9 x86_64 (Linux kernel 5.15)

Nvidia Driver Version: 510.73.08

CUDA Version: 11.6

Factorial Experiments

The raw factorial experiment data is shown in Table S4. Here, we have tested all these five input features ($H'_{\mathrm{bond},i}$, $H'_{\mathrm{atom},i}$, $H'_{\mathrm{self},i}$, $H'_{\mathrm{MNE},i}$ and $H'_{\mathrm{SEN},i}$, we shall call them F1, F2, F3, F4 and F5 respectively in the later paragraphs) in either disable (marked as 0, set to 0) and enable (marked as 1, keep original value) status, with the DDEC dataset and the 5x50 neural network (batchs=128). For example, for model "DDEC_FE_00011", features F1, F2 and F3 will be set to 0 and features F4 and F5 will keep their original value when input in both training and testing processes.

Here we calculated both the MAE (mean absolute error) and RMSE under different circumstances. As the loss function used in NN training is MAE, we then choose MAE for following analysis. We followed the following formula to calculate the interaction between factors:

$$\text{Interaction}_{ij} = \mu_{h_i h_j} + \mu_{l_i l_j} - \mu_{h_i l_j} - \mu_{l_i h_j}, \tag{4}$$

where i and j each is one of the factors and $i \neq j$, and μ is the average MAE in these three tests. h_i stands for the condition when factor i is enable and l_i is when i is disable. The calculated interaction matrix is:

Table S2: Interaction Matrix

	F1	F2	F3	F4	F5
F1	———	0.02020	-0.01103	-0.02080	0.01335
F2	———	———	-0.00739	-0.02840	0.01646
F3	———	———	———	0.00570	0.03025
F4	———	———	———	———	-0.00550
F5	———	———	———	———	———

For the mean effect of each factor, we use:

$$\text{Effect}_i = \mu_{h_i} - \mu_{l_i}. \tag{5}$$

So the mean effect of each factor can be calculated as:

Table S3: Table of Mean Effect

	F1	F2	F3	F4	F5
**Mean Effect**	0.00120	-0.00080	-0.02007	-0.02095	-0.05345

It can be observed that all these five features have interactions to others and can affect the prediction error. From Table S2, we can see there are strong interactions between F1 and F2, F1 and F4, F2 and F4, and F3 and F5. Table S3 shows that F3, F4 and F5 have stronger mean effect on the prediction error. It can be concluded that all of these five factors have significant contribution to the accuracy of final prediction.

Table S4: Factorial Experiment Raw Data

**Model**	**test_1**		**test_2**		**test_3**	
	MAE	RMSE	MAE	RMSE	MAE	RMSE
DDEC_FE_00000	0.182985	0.256157	0.182955	0.256062	0.182882	0.255826
DDEC_FE_00001	0.11444	0.168789	0.115531	0.167496	0.114881	0.167073
DDEC_FE_00010	0.141524	0.214434	0.156863	0.207528	0.13119	0.205818
DDEC_FE_00011	0.047824	0.071054	0.050304	0.075557	0.057385	0.088112
DDEC_FE_00100	0.168933	0.241049	0.168926	0.242032	0.170278	0.240173
DDEC_FE_00101	0.122074	0.176235	0.113262	0.172378	0.120806	0.181175
DDEC_FE_00110	0.106063	0.172062	0.108881	0.181664	0.08822	0.155635
DDEC_FE_00111	0.049534	0.072191	0.042921	0.06692	0.045614	0.067594
DDEC_FE_01000	0.170218	0.245686	0.170914	0.246125	0.170702	0.246598
DDEC_FE_01001	0.112935	0.178926	0.116763	0.183241	0.115916	0.182624
DDEC_FE_01010	0.130098	0.213132	0.107443	0.17857	0.128597	0.211475
DDEC_FE_01011	0.046286	0.070452	0.04622	0.070551	0.043834	0.069449
DDEC_FE_01100	0.154255	0.232107	0.154472	0.234274	0.153202	0.229011
DDEC_FE_01101	0.103488	0.163212	0.111513	0.17314	0.107722	0.168376
DDEC_FE_01110	0.079564	0.141528	0.08205	0.141866	0.083739	0.144886
DDEC_FE_01111	0.045961	0.069487	0.043987	0.069154	0.04266	0.066044
DDEC_FE_10000	0.170959	0.24898	0.17174	0.247678	0.171696	0.248459
DDEC_FE_10001	0.12225	0.189323	0.122301	0.187317	0.11897	0.184224
DDEC_FE_10010	0.133914	0.206018	0.115384	0.1802	0.124977	0.187899
DDEC_FE_10011	0.052603	0.080767	0.051399	0.077703	0.049595	0.07468
DDEC_FE_10100	0.147469	0.232026	0.151796	0.231911	0.148715	0.231957
DDEC_FE_10101	0.096524	0.157471	0.098503	0.161814	0.09966	0.161735
DDEC_FE_10110	0.099805	0.165318	0.096876	0.1655	0.09464	0.159751
DDEC_FE_10111	0.039843	0.059057	0.045526	0.06587	0.045505	0.065537
DDEC_FE_11000	0.216486	0.292597	0.258398	0.324011	0.267535	0.325066
DDEC_FE_11001	0.155527	0.202688	0.153883	0.196417	0.144166	0.189579
DDEC_FE_11010	0.100978	0.16545	0.073093	0.126257	0.080736	0.134493
DDEC_FE_11011	0.0478	0.07122	0.049823	0.074254	0.045714	0.071007
DDEC_FE_11100	0.138173	0.22849	0.133899	0.215842	0.140153	0.227923
DDEC_FE_11101	0.187789	0.228873	0.141372	0.204438	0.120644	0.183977
DDEC_FE_11110	0.070349	0.11832	0.064038	0.114235	0.071777	0.126298
DDEC_FE_11111	0.045243	0.066701	0.04335	0.066182	0.042973	0.062951

References

(1) Gilmer, J.; Schoenholz, S. S.; Riley, P. F.; Vinyals, O.; Dahl, G. E. Message passing neural networks. *Machine learning meets quantum physics* **2020**, 199–214.

(2) Cho, K.; Van Merriënboer, B.; Bahdanau, D.; Bengio, Y. On the properties of neural machine translation: Encoder-decoder approaches. *arXiv preprint arXiv:1409.1259* **2014**,

(3) Group, N. L. C. R-NET: Machine Reading Comprehension with Self-matching Networks. 2017.
